# Effects of Sugar on the Teeth

**Published:** 1865

**Authors:** 


Effects of Sugar on the Teeth.—Drs. Paolo Manlegazza and Labus, of the University of Pavia, have recently undertaken a series of experiments for investigating the action of sugar on the teeth, and report that sugar (as sugar) does not exercise any chemical action upon the teeth, and that it does not predispose to caries; but only affects the teeth when it has undergone the acetic or lactic fermentation.

